# IgM and C3 Deposition in Primary Focal Segmental Glomerulosclerosis (FSGS): A Clinical and Histopathological Spectrum

**DOI:** 10.7759/cureus.37346

**Published:** 2023-04-09

**Authors:** Faizan Amer, Madiha Syed, Aurangzeb Afzal, Mudassar Hussain, Usman Hassan, Shaarif Bashir, Maryam Hameed, Sheeba Ishtiaq

**Affiliations:** 1 Pathology, Shaukat Khanum Memorial Cancer Hospital and Research Centre, Lahore, PAK; 2 Nephrology, Services Hospital Lahore, Lahore, PAK; 3 Pathology, Shaukat Khanum Memoiral Cancer Hospital and Research Centre, Lahore, PAK; 4 Pathology, Ghulab Devi Teaching Hospital, Lahore, PAK

**Keywords:** immune deposition, prognosis, c3, igm, focal segmental glomerulosclerosis (fsgs)

## Abstract

Focal segmental glomerulosclerosis (FSGS) is a common renal disorder, characterized by progressive segmental sclerosis of renal glomeruli and clinical symptoms corresponding to proteinuria. Classically, it is not considered to be an antibody-mediated disease, however, IgM and C3 deposition may be seen in a subset of cases of FSGS. The impact of this immune deposition on histopathological features in renal core biopsies, on the urinary biochemical parameters, and the clinical outcomes, has not been previously investigated in our population. The aim of this study is to analyze the aforementioned parameters in patients with primary FSGS having antibody deposition as compared to those who do not have any antibody deposition.

Some 155 patients diagnosed with FSGS were retrospectively enrolled in our study. The renal biopsies were reviewed for histopathological features and immunofluorescence (IF) findings of IgM and C3 glomerular deposition. These histological features were then compared with the biochemical parameters as well as the clinical outcomes of patients. The patients were assigned to Groups 1 and 2 based on the IF findings.

The IgM and/or C3 glomerular deposition had a low incidence in patients with primary FSGS in our study (28.3%). Patients having IgM and C3 co-deposition had a significantly longer time duration since the onset of their clinical symptoms; active disease duration (42 months vs 22 months, p=0.049). The mean pre-treatment serum creatinine of patients with IgM and C3 co-deposition was 6.00 mg/dL as compared to 3.29 mg/dL in patients with no immune deposition (p=0.037). The immune deposition was associated with higher rates of segmental and global glomerulosclerosis, but this finding along with other evaluated histological parameters did not show statistical significance. The number of patients having IgM and/or C3 deposition and with active steroid use/renal dialysis was similar to patients having no IgM and/or C3 deposition.

The IgM and/or C3 deposition in FSGS has a low incidence within and is not associated with any significant differences in histological parameters on renal core biopsies of patients from the Pakistani population. IgM and/or C3 deposition is also associated with a significantly longer duration of active disease and these patients may present with higher pre-treatment serum creatinine. Other biochemical parameters and clinical outcomes appear comparable between the groups based on the available clinical data.

## Introduction

Focal segmental glomerulosclerosis (FSGS) [1] is a common renal disorder that clinically presents as nephrotic syndrome and is characterized by a specific pattern of glomerular injury. Focal segmental sclerosis of glomeruli is seen on light microscopy and effacement of foot processes of podocytes can be seen on electron microscopy. Several morphological patterns of FSGS [2] are also identified with prognostic significance. FSGS is not a distinct clinical disease, rather it encompasses a large group of disorders that may result in this pattern of glomerular injury. The incidence of FSGS is on the rise and it is now one of the most common causes of steroid resistant-nephrotic syndrome in pediatric [3-4] and adult age groups [5], both worldwide and in the Pakistani population. It also has frequent progression to end-stage renal disease, with the literature citing [6] the African population as being particularly susceptible. The pathogenesis of FSGS is extremely complex with studies showing numerous underlying mechanisms [7-9] such as circulating permeability factors, decreased nephron mass, podocyte injury, and podocyte depletion. The disease may occur idiopathically (primary FSGS) or secondarily to diverse etiologies [10-11] such as viruses, drugs, and genetic syndromes (secondary FSGS).

The diagnosis of FSGS requires the integration of clinical history, histopathology findings in a renal core biopsy, immunofluorescence (IF), electron microscopy, and recently, genetic studies. IF [12] is a valuable diagnostic modality that has become a central pillar when it comes to the diagnosis of renal disease. Many renal disorders are caused by the deposition of various antibodies, immune complexes, and immunological factors in the glomeruli and other renal structures. Detection of these deposited antibodies and immune factors greatly aids in the diagnosis of renal disorders. FSGS may be associated with IgM and/or C3 deposition in the glomeruli in a subset of patients. Studies [13-14] show that such patients may present with a worse biochemical profile, may show poor response to conventional immunosuppressive therapy, and may be associated with a worse clinical outcome.

The aim of this study is to evaluate histopathological changes on renal core biopsies as well as clinical parameters of patients with FSGS and to compare whether the deposition of IgM, C3, or both are associated with a poor clinical outcome, as opposed to patients having renal biopsies with a negative IF result.

## Materials and methods

This retrospective and non-interventional study was conducted in the Histopathology Department of Shaukat Khanum Memorial Cancer Hospital and Research Centre, Lahore, Pakistan. The study was approved by the Hospital’s Internal Review Board and a waiver of informed consent was obtained. The Hospital Information System was used to retrieve all cases of FSGS diagnosed at the hospital between 2016 and 2020. According to the definition of FSGS as per the Colombia classification, 350 cases of FSGS were diagnosed in our hospital during this time period.

In our laboratory, all renal cores are received in 10% buffered formalin for processing for light microscopy and in normal saline for IF. For light microscopic analysis, multiple thin sections (three microns) are taken and stained with hematoxylin and eosin (H&E) as well as with special stains including periodic acid Schiff (PAS), Jones Methenamine Silver (JMS), and Masson’s Trichrome. For IF, the renal cores received in normal saline are placed in Michel’s Transport Medium for 1 h, followed by a wash in a buffer solution. The cores are then frozen at -20°C to -22°C and thin sections are taken. A panel of antibodies (IgG, IgM, IgA, C3, and C1q) is then applied by using a Dako Link 48 Autostainer (Agilent, Santa Clara, CA) at a dilution of 1:20. The prepared slides are then mounted with a fluorescent mounting media and are stored at 2°C-8°C in a refrigerator for subsequent microscopic analysis. Our laboratory neither has the facilities to process specimens for electron microscopy nor do we have an electron microscope available to us.

Cases in which FSGS were the only pattern of glomerular injury, had classic histological features, satisfactory results of special stains, and adequate clinical information was included in the study. All cases in which FSGS was associated with a secondary glomerular injury pattern, unsatisfactory results of special stains, inadequate clinical data, or a clear association with a cause of FSGS (secondary FSGS) were excluded from the study. Additionally, all cases of FSGS diagnosed in post-renal transplant patients were excluded from the study. The distinction between primary and secondary FSGS was made based on the clinical history, as we did not have access to electron microscopy.

Of the 350 cases of FSGS that were retrieved, 86 cases were excluded as these were biopsies from post-renal transplant patients or from patients with long-standing diabetes or hypertension prior to the development of renal disease. A further 108 cases could not be included in the study as they had either been lost to clinical follow-up or had clinical data insufficient for inclusion in the study.

The H&E as well as Special Stain slides were retrieved from the storage and were reviewed by two renal pathologists. We did not encounter any discrepancies in the reported diagnosis upon review. The IF slides were not reviewed; however, a positive IF pattern was defined as the deposition of either IgM, C3, or both. IF staining was graded as negative, mild (1+), moderate (2+), and strong (3+). The histopathological variants of FSGS were defined according to the Columbia classification (NOS, Tip, Perihilar, Cellular, and Collapsing). The chronic tubulointerstitial injury (tubular atrophy and interstitial fibrosis, IFTA) was graded semi-quantitatively as mild (0 to 25% of interstitium involved), moderate (25%-75%), and severe (>75%). The intensity of PAS staining in the sclerotic areas of the glomeruli was scored as 1-3. The vascular changes were divided into atherosclerosis and arteriosclerosis and were then graded as mild, moderate, and severe.

For the purpose of comparison, the cases with a positive IF pattern were labeled as Group 1 while the patients with a negative pattern were labeled as Group 2. Group 1 was further sub-stratified into (IgM+ C3-), (IgM+ C3+), and (IgM- C3+) groups for further analysis where required.

Data collection and statistical analysis

The clinical and laboratory data were obtained from hospital records and through telephonic conversations with the patients. Questions were regarding clinical and biochemical parameters such as symptoms, time since onset of symptoms, serum creatinine, serum urea, 24-h urinary protein, the current state of the patient (alive or deceased), steroid therapy, and renal replacement therapy. The data on biochemical parameters that could be obtained was from the pre-treatment stage. The post-treatment data could either not be reliably obtained or was not sufficient for inclusion in the study. The exact dosage and duration of steroid administration were also not available to us.

Nephrotic range proteinuria was defined as urinary protein excretion greater than 3.5 g/24 h. Response to treatment or ‘Clinical Remission’ was defined on the basis of the resolution of clinical symptoms and cessation of steroid usage/renal replacement therapy. Incomplete treatment response was defined as the continued usage of steroids and/or renal replacement therapy.

The IBM SPSS version 26 (IBM, Armonk, NY) was utilized for statistical analyses. Mean ± standard deviation/median (range) was employed to summarize quantitative data (age, etc.), whereas frequencies and percentages were used to organize qualitative data; sex etc. Independent-t-test and a one-way analysis of variance (ANOVA) test were used to calculate the mean difference. p-value ≤0.05 was considered statistically significant.

## Results

Some 155 cases of FSGS met the inclusion criteria and were included in the study. The overall male to female ratio was 1.9:1, which is statistically significant (p=0.043). The mean age of male patients was 30.7 years (standard deviation, SD = 14.5 years) and of female patients was 29.5 years (SD = 14.6 years). After division into groups based on IF results, Group 1 had a M:F ratio of approximately 1.6:1 (27M:17F) and a mean age of 29.6 years while Group 2 had a M:F ratio of 2:1 (M=76, F=35) and a mean age of 32.1 years. There was no significant difference in the mean age or the sex distribution between the two groups (p=0.369 and 0.254, respectively).

The majority of the patients in our study presented with the clinical symptoms of pedal/periorbital edema (n=118, 76.1%) and proteinuria (n=24, 15.5%). Some 51 patients had a complaint of hematuria but a clear distinction between gross hematuria and microscopic hematuria could not be established from the available clinical data. There was also no statistically significant difference in incidence between the two groups (p=0.467). Patients having IgM and C3 co-deposition were found to have a significantly greater mean time since the start of their clinical symptoms and disease duration (42 months) as compared to patients with a negative IF pattern (22 months, p=0.049). Patients in Group 1 who had IgM and C3 co-deposition had a mean pre-treatment serum creatinine value of 6.00 mg/dL as compared to 3.29 mg/dL for Group 2. The 24-h urinary protein was also found to be higher in Group 1, with patients having isolated C3 deposition having the highest rate of protein excretion per day (4.87 g/day), followed by 4.53 g/day for patients with IgM and C3 co-deposition. In comparison, Group 2 had a protein excretion of 4.38 g/day. However, this finding was not found to be statistically significant (p=0.144). Interestingly, mean serum urea levels were found to be higher in Group 2 (103 mg/dL) as compared to Group 1 (80 mg/dL, p=0.032). Table 1 summarizes the demographics and clinical data.

**Table 1:** Demographics, clinical symptoms, and biochemical parameters of patients with FSGS. FSGS, focal segmental glomerulosclerosis; SD, standard deviation

Characteristics	Group 1 (IgM+/-­­ or C3+/-)		Group 2 (IgM- and C3-)	p value
Male/female (n)	27/14	76/35		
Age, years, mean (SD)	29.6 (15.3)	32.1 (14.1)		0.369
Hematuria (%)	12 (27.3%)	39 (35.1%)		0.467
Pedal edema/generalized edema, n (%)	31 (70%)	87 (78%)		Not available
Proteinuria, mean	4.50	4.38		0.144
Serum creatinine (mg/dL)	6.00	3.29		0.037
Serum urea (mg/dL)	80	103		0.032

Histopathology and immunofluorescence

**Figure 1:**
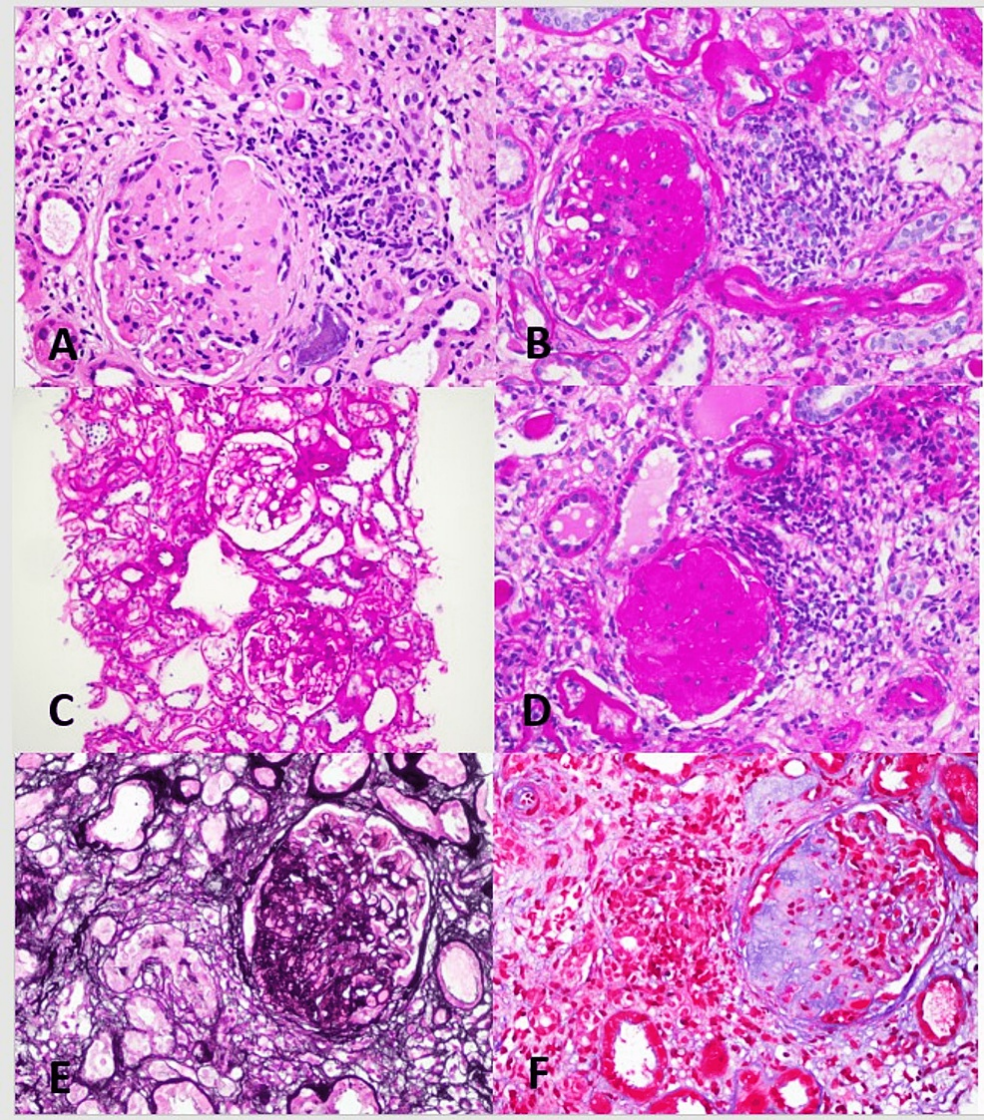
FSGS. (A) The right half of this glomerulus is involved by dense mesangial sclerosis and adhesion to the Bowman’s capsule. The sclerosis obliterates the capillary lumina. The sclerosis is characteristically segmental in this glomerulus with sparing of the left side of the glomerulus (H&E stain). (B) The sclerosed areas appear brightly eosinophilic on PAS special stain. (C) The focal nature of the disease is highlighted in this example as the glomerulus on the top appears normal while the glomerulus on the bottom is involved by sclerosis and adhesion to the Bowman’s capsule (PAS stain). (D) FSGS may eventually progress to global glomerulosclerosis, which is characterized by the complete obliteration of the glomerulus by dense fibrous tissue (PAS stain). The glomerular sclerosis is highlighted by Jones Methenamine Silver (E) and Trichrome (F) special stains. FSGS, focal segmental glomerulosclerosis

The most common histological feature of FSGS in our cases was the focal and segmental sclerosis of renal glomeruli. The sclerotic areas were characterized by dense, acellular matrix material involving the glomerular capillary loops with their obliteration, as well as involving the mesangium. Global glomerulosclerosis was also seen. The histological features are highlighted in Figure 1.

**Figure 2:**
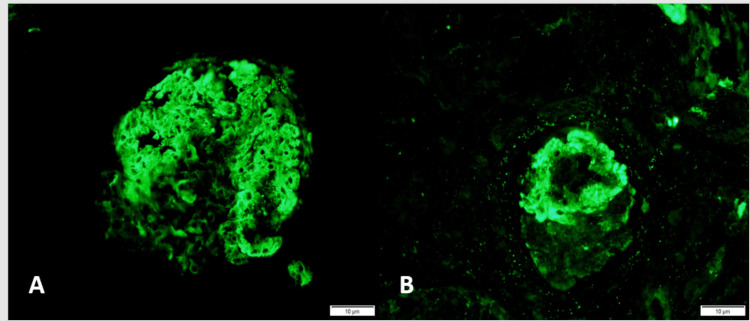
Immunofluorescence findings in FSGS. A subset of cases of FSGS show segmental deposition of IgM and C3. There is moderate segmental glomerular tuft staining by IgM (A) and strong staining by C3 (B) in this example. FSGS, focal segmental glomerulosclerosis

Some 44 of 155 cases (28.3%, Group 1) had a positive IF pattern. Isolated IgM deposition was the most common finding (n=24,15.5%), followed by deposition of both IgM and C3 (n=12,7.7%). Some eight cases had deposition of C3 alone (n=8,5.2%). The remaining 111 (71.6% Group 2) cases had either a negative IF pattern or had deposition of other antibodies which were not a part of our study. The IgM and C3 staining were recorded as mild (IgM=58% of cases, C3=65% of cases), moderate (IgM=36%, C3=30%), and strong (IgM=6%, C3=5%), segmental glomerular staining. IF findings are shown in Figure 2.

The mean number of glomeruli involved by segmental sclerosis was greater for Group 1 (n=4.0) as compared to Group 2 (n=3.5). Patients with IgM and C3 co-deposition had the highest mean number of segmentally sclerosed glomeruli (n=4.5) followed by patients with isolated C3 deposition (n=4.0). These findings did not turn out to be statistically significant. Group 1 had 23 cases (52%) with global glomerular sclerosis while Group 2 had 50 cases (45%). The mean number of glomeruli involved by global sclerosis per renal biopsy was 1.9 (SD=4.0) for Group 1 and 1.7 (SD=3.0) for Group 2. Although this finding was not statistically significant (p=0.802), patients having IgM and C3 co-deposition were found to have twice the mean number of globally sclerosed glomeruli (n=3.3) as compared to patients having a negative IF pattern (n=1.7).

The most common histological variant of FSGS in our study was the NOS variant (n=111,71.6%), followed by the tip variant (n=34,22%). The incidence of various patterns is summarized in Table 2.

**Table 2:** Frequency of various histopathological variants of FSGS in Groups 1 and 2. FSGS, focal segmental glomerulosclerosis

	Group 1 (%)	Group 2 (%)
Cellular	5	4
Collapsing	0	2
Perihilar	5	0
Tip	20	22
Nos	70	72

**Figure 3:**
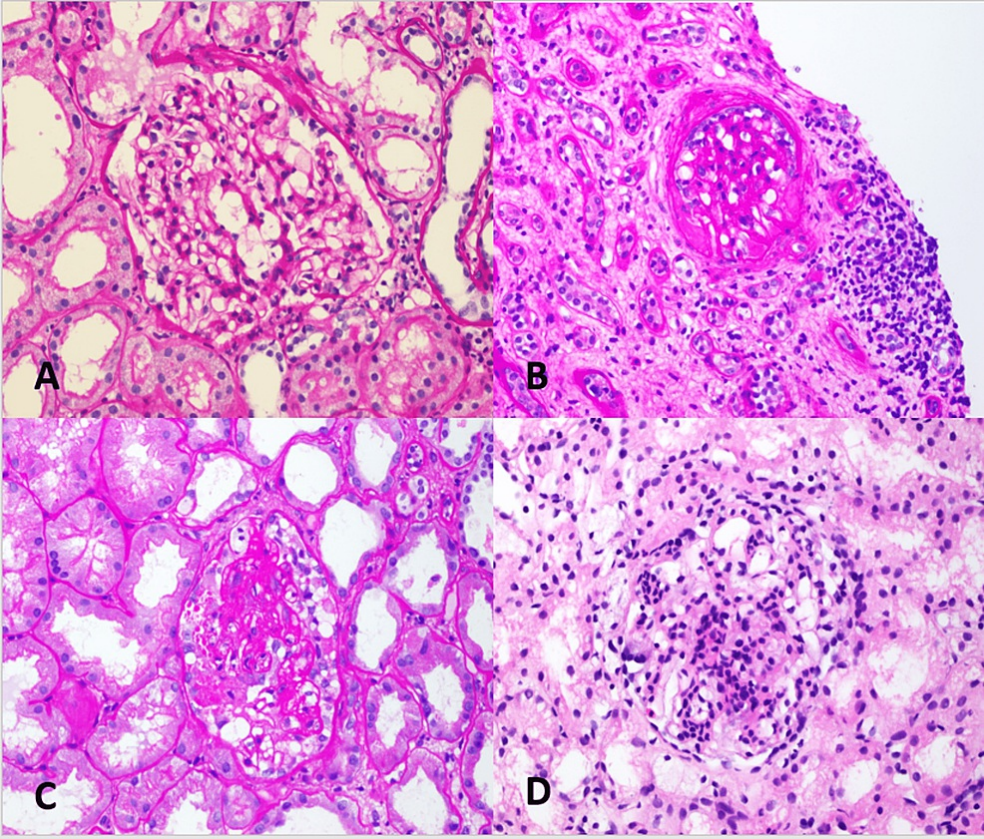
Morphological spectrum and histological variants of FSGS. (A) The tip variant of FSGS is characterized by a small segmental lesion involving the glomerulus adjacent to the tubular pole. There is endocapillary foam cell accumulation and the glomerulus appears to prolapse into the proximal tubule. The podocytes may appear confluent with the proximal tubular cells (PAS stain). (B) The Perihilar variant of FSGS shows segmental sclerosis of the glomerulus predominantly at the vascular pole (PAS stain). (C) FSGS, collapsing variant. This example shows a global collapse of the glomerular capillaries coupled with marked hyperplasia and swelling of overlying parietal epithelial cells (PAS stain). (D) In the cellular variant of the FSGS, there is a segmental expansion of the glomerular tuft due to endocapillary hypercellularity, infiltration by lymphocytes, and foam cells coupled with variable epithelial cell hyperplasia (H&E stain). FSGS, focal segmental glomerulosclerosis

NOS was the most common pattern in both groups and there was no significant difference in the incidence of histological patterns between the two groups (p=0.299). Further details and photomicrographs of various patterns of FSGS are provided in Figure 3.

The PAS staining in the sclerotic areas was intense (score 3) in the majority of the cases (n=108,69.7%) and it did not correlate with IF result (p=0.485). Interstitial fibrosis and tubular atrophy (IFTA) was graded as mild in 43.2% (n=67) and moderate in 31% (n=48) of cases, with no statistically significant difference between the groups (p=0.289). Interstitial inflammation comprised lymphocytes that were observed in 115 (76%) cases with 69 cases (44.5%) having mild inflammation, 38 cases (24.5%) having moderate inflammation, and eight cases (5.2%) having severe interstitial inflammation. The finding was not found to be statistically different between the two groups. (p=0.107). Mild to moderate atherosclerosis (40.6%) and arteriosclerosis (45.8%) were seen in the renal biopsies. These vascular changes did not correlate with the IF findings (p=0.294 and p=0.314, respectively). The histological parameters are summarized in Table 3.

**Table 3:** Histopathological characteristics of FSGS patients with and without IgM/C3 deposition (N/A). FSGS, focal segmental glomerulosclerosis; N/A, not applicable

Characteristics	Group 1 (IgM+ and/or C3+)	Group 2 (IgM- and C3-)		p-value
Segmental glomerulosclerosis, mean no. glomeruli	4.0		3.5	0.986
Global glomerulosclerosis, n/mean (%)	23/1.9(52)		50/1.7(45)	0.803
Interstitial fibrosis and tubular atrophy, n (%)				0.289
Absent	30(27)		7(15.9)	
Mild	24(54.5)		43(38.7)	
Moderate	12(27.3)		36(32.4)	
Severe	1(2.3%)		2(1.8)	
IgM deposition, n (%)				N/A
Mild	21(58)		N/A	
Moderate	13(36)		N/A	
Strong	2(6)		N/A	
C3 deposition, n (%)				N/A
Mild	13(65)		N/A	
Moderate	6(30)		N/A	
Strong	1(5)		N/A	
Interstitial Inflammation, n (%)				0.107
Nil	17(20.7)		23(38.6)	
Mild	18(45.9)		51(40.9)	
Moderate	8(27.0)		30(18.2)	
Severe	1(6.3)		7(2.3)	
Arteriosclerosis, n (%)				0.314
Nil	20(45.5)		51(45.9)	
Mild	10(22.7)		18(16.2)	
Moderate	13(29.2)		30(27.0)	
Severe	1(2.3)		12(10.8)	
Atherosclerosis, n (%)				0.325
Nil	17(38.6)		61(55.0)	
Mild	12(27.3)		24(21.6)	
Moderate	10(22.7)		17(15.3)	
Severe	5(11.4)		5(8.1)	

Clinical outcomes

The majority of the patients in both groups were still regularly using steroids at the time of the commencement of this study. A higher number of patients in Group 1 had reported active steroid use (n=35,79.5%) as compared to patients in Group 2 (n=77,69.5%). Some eight (18.2%) patients in Group 1 and 23 (20.7%) patients in Group 2 were on renal replacement therapy at the time of the commencement of the study. A positive IF result did not appear to correlate with either increased use of steroids (p=0.470) or the continued requirement of renal replacement therapy (p=0.772). Some 21.6% of the patients in Group 1 had reported resolution of their clinical symptoms, no active steroid use, or a requirement for renal replacement therapy at the time of commencement of the study as compared to 20.5% in Group 2. Some 16.2% of the patients in Group 1 had died due to disease-related complications as compared to 13.6% in Group 2. These findings were also comparable between the two groups. Table 4 summarizes these clinical parameters.

**Table 4:** Clinical parameters of FSGS patients in relation to IgM and C3 deposition. Clinical remission was defined as the resolution of clinical symptoms coupled with no active steroid use or renal dialysis.

Characteristics	Group 1 (IgM+and/or C3+)	Group 2 (IgM- and C3-)	p value
Active steroid use, n (%)	35(79.5)	77(69.5)	0.470
Ongoing renal replacement therapy, n (%)	8(18.2)	23(20.7)	0.772
Disease-related mortality, n (%)	6(13.5)	18(16.2)	0.708
Clinical remission, n (%)	9(20.5)	24(21.6)	0.967

## Discussion

Focal segmental glomerulosclerosis is now one of the most common causes of nephrotic syndrome in pediatric and adult age groups [4, 15-16] within the Pakistani population as shown by several studies. The prognosis is variable, with some patients going into remission while others progress to end-stage renal disease. This progression is also reflected in histology, with an increasing number of glomeruli becoming globally sclerosed. FSGS may be associated with irregular deposition of IgM or C3 or both in the sclerotic areas in a number of cases. It is proposed that various insults may generate neo-epitopes within the glomeruli in a subset of patients of FSGS with which IgM and C3 may bind [17-19] and then activate the complement system. The complement system is a group of serum proteins and is a part of the innate immune system. Activation of the complement proteins initiates various protective mechanisms; however, over-activation can also have deleterious effects, especially on the kidney. The system is activated by classical, alternative, and lectin pathways, which initiate inflammation, the release of chemotactic molecules, and the formation of a membrane attack complex. Serum C3 is a good indicator of complement activation. The IgM/C3 mediated complement damage has been associated with a worse clinical outcome [13-14] in several studies.

Our study is a retrospective analysis of FSGS patients with a comparison of their histopathological and clinical features in relation to IgM and/or C3 deposition within the glomeruli. To the best of our knowledge, there has been no other study conducted on our population that has analyzed patients of FSGS in relation to these IgM and/or C3 depositions. We observed a Male predominance for incidence of FSGS in both of our groups, with a M:F ratio of almost 2:1. This result was similar to comparable studies in the Pakistani, Indian, and Chinese populations. The mean age of FSGS patients in our study was slightly higher than the one reported by Swarnalatha et al. [2] in the Indian population (24 years) and lower than that reported by Mirioglu et al. [13] in the Turkish population (36 years). We did not observe any significant difference in the mean age of patients when stratified into Groups 1 and 2 or when further stratified based on the deposition of IgM, C3, or both. These findings were similar to the one reported by Mirioglu et al. [13] but were in contrast to findings reported by Zhang et al. [14] who had reported a much younger age of patients having IgM and C3 deposition (24 years) as compared to ones with no deposition (46 years). These findings show that there may be significant differences in the mean age of FSGS patients in certain populations. 

In our study, 28.3% of FSGS patients in our study had a positive IF pattern with isolated IgM deposition being the most common (15.5%), followed by IgM and C3 co-deposition (7%). The staining was mostly of mild to moderate intensity. This finding of IgM and C3 co-deposition also indicates that IgM may not have a role in the onset of the disease but rather is deposited after a glomerular injury has occurred due to other reasons. Nasir et al. [16] have reported similar results in their study on the IF pattern of FSGS in the Pakistani population. Our results were in contrast to studies in other populations. Zhang et al. [14] reported a much percentage of IgM deposition (54.7% of their patients) in the Chinese population while Mirioglu et al. [13] reported 51.1% within the Turkish population. These findings show that there are significant differences in the incidence of IgM and/or C3 deposition in patients with primary FSGS of different ethnicities.

Patients with IgM and C3 co-deposition had a higher mean number of glomeruli involved in both segmental and global glomerular sclerosis. However, none of the histological findings in our study were found to have statistical significance. Interstitial inflammation, fibrosis, tubular atrophy, and vascular abnormalities were all found to be comparable between the two groups. These findings suggest that histological changes cannot reliably predict which cases will show glomerular deposition of IgM and/or C3 and that glomerular deposition of IgM and/or C3 may not be associated with any specific histological changes. Mirioglu et al. [13] reported similar findings of histological parameters in their study, however, they reported a statistically significant increase in segmental glomerular sclerosis in patients having IgM and C3 deposition. The histological variants of FSGS are also not associated with any particular IF pattern.

We found similar presenting clinical symptoms in both groups, with nephrotic range proteinuria and generalized edema being the most common symptoms. Hematuria was reported by one third of the patients but it did not correlate with the IF findings. Patients in Group 1 had a statistically significant increase in mean serum creatinine value as compared to Group 2 (6 and 3 mg/dL, respectively, p=0.037). Proteinuria was also higher in Group 1, with patients having isolated C3 deposition and IgM and C3 co-deposition having the highest levels of proteinuria, however, this finding was not statistically significant. Although these findings were not statistically significant in our study, they still corroborate previous studies that suggest that not only IgM but also C3 may be an important factor in initiating complement-mediated injury [19] after its deposition within the glomeruli. A low serum C3 has also been shown to be an independent risk factor for disease progression [20] in FSGS and in our study, patients with isolated C3 deposition also had the second highest number of segmentally sclerosed glomeruli. The cause of higher serum urea levels in patients with no immune deposition is uncertain.

Our study did not find significant differences in the clinical parameters of patients with primary FSGS. Both groups reported comparable results for the active use of steroids (p=0.470) as well as for the continued requirement of renal replacement therapy (p=0.772). The response to treatment also did not differ significantly between the two groups as a similar percentage of patients in both groups had reported clinical remission. These results were in contrast to clinical studies conducted by Mirioglu et al. [13] and Zhang et al. [14], who had reported that IgM and C3 co-deposition is an independent risk factor of poor renal outcome in patients with FSGS. In these studies, patients with IgM and C3 co-deposition had higher levels of proteinuria, decreased estimated glomerular filtration rate (eGFR), refractoriness to immunosuppressive therapy, and an overall poor renal outcome in such patients.

This is a single-center study on the histopathological and clinical spectrum of IgM and/or C3 deposition in patients with FSGS. To the best of our knowledge, this is the first study conducted on the Pakistani population which analyzes this disease and the role of IgM and/or C3 deposition in renal glomeruli. One of the limitations of our study is that a cause-effect relationship cannot be reliably made due to the retrospective-observational nature of the study. We also neither had complete clinical information of our patients nor the exact duration or dosage of steroid usage, thus we could not statistically assess the treatment response of FSGS patients having IgM and/or C3 deposition. As previously mentioned, electron microscopy was not available to us, thus the distinction between primary and secondary FSGS was based on clinical history.

Despite these limitations, we showed that although there were no statistically significant histological changes attributable to IgM and/or C3 deposition, such patients did have higher rates of segmental and global glomerulosclerosis coupled with a higher level of proteinuria and serum creatinine. As we have shown that IgM and/or C3 deposition has a low incidence in our population, validation from other multicenter studies with larger cohorts is required to establish the definite prognostic role of IgM and/or C3 deposition in FSGS patients in the Pakistani population. The retrospective nature of the study and the limitations of available clinical data should encourage other investigators to conduct a prospective trial to answer the questions raised by our study. The lack of electron microscopy adds another limitation to our study but the curiosity created by the unexpected results may be instrumental in planning a study with electron microscopy. This study may not have all the answers but is able to raise sufficient questions to cultivate ideas of prospective research on the topic in the minds of regional investigators.

## Conclusions

The IgM and/or C3 deposition in FSGS has a low incidence within the Pakistani population and is not associated with any significant differences in histological parameters on renal core biopsies. It is also associated with a significantly longer duration of active disease and these patients may present with a higher pre-treatment serum Creatinine. Larger, multicenter studies are required on our population to analyze clinical parameters in order to assess any statistically significant difference.
